# Novum Organum Medicorum; a New Medical Logic

**Published:** 1826-10-01

**Authors:** 


*Novum Organum Medicorum; a New Medical Logic, or the Art of Thinking and Right Reasoning applied to Practical Medicine; exhibiting the Principles advanced in a larger Work under the same Title.* By VINZENZO LANZA, M.D. Professor of Clinical Medicine in the Ospidale della Pace, at Naples. Translated from the Italian; By C. STORMONT, M.D. London; Samuel Highley, 174, Fleet Street, and Webb Street, St. Thomas's Hospital, 1826. Pp. 284.

[Concluded from page 149.]

UNDER the denomination of general morbid forms, our author first treats of inflammation—obstruction—and congestion, which, he says, are as similar in their phenomena as they are analogous in their nature.

*Phenomenology.* Swelling, redness, heat, and pain, are symptoms common to all these forms of disease, and their diverse combinations produce the distinctions of name and appearance. When the heat is ardent, the pain acute, the redness bright, and the swelling elastic, the affection is termed inflammation. When the swelling is great and firm, the heat slight, the redness obscure, and the pain dull, the affection is called obstruction. Congestion is a collection of humours in a part, producing a soft swelling with a dull pain, some heat and redness. There are intermediate forms of disease between these now described. For example, between inflammation and obstruction, there may be a swelling characterized by hardness and little heat, and which may be termed either a slow inflammation or a hot obstruction, as it inclines to the one or other of these distinct forms. A swelling that is very hot and rather soft may be called a hot congestion, inflammatory engorgement, or congestive inflammation. An obstruction may be humid or dry; in the former, the part is rather large than hard; in the latter, on the contrary, the hardness is more striking than the swelling, and in some cases of dry obstruction, the bulk of a part may be actually less than in the healthy state, while the hardness is very great. Congestions are also termed sanguineous, bilious, phlegmatic, &c. according as blood, bile, or lymph predominate in the afflux of humours.

The visible changes which occur in the structure of parts under these forms of disease, are an accumulation in the cellular tissue of the affected organ, of a fluid more dense than that thin lymph which naturally moistens the part in the healthy state. In inflammation, this humour coagulates; in obstruction, it becomes rather firm and dry; in congestion, it remains liquid, either white, yellow, or sanguinolent. In all these affections the vessels of the part and those adjacent are enlarged, the smaller vessels, which are not visible in the healthy state, are filled with blood and become visible; and, lastly, in the opinion of some authors, there spring up and become developed new vessels, which the organ did not originally possess. In inflammations, accompanied with fever, the blood drawn from the veins is speedily replaced, it has little or no serum, and becomes covered with a whitish crust, sometimes of a yellowish or greenish hue.

As inflammation is acute when it possesses the true inflammatory characters, so it is of a chronic nature when it verges towards obstruction or congestion. In like manner a true obstruction is chronic, and in proportion as it deviates towards being acute, does it participate of the nature of inflammation and congestion. Congestion, when acute, participates of the nature of inflammation; when chronic, it inclines towards obstruction.

Inflammation, obstruction, and congestion, have most generally a continued course, but it is not uncommon, especially when they are elements of a periodical disease, for them to suffer periodically some increase and diminution.

*Etiological Pathology.* A disposition to inflammation, obstruction, and congestion, exists in the body, and may be said to consist in the whole body, or some particular organ or part of it, being slightly, and in an occult manner, predisposed; so that, on the application of any slight exciting cause, the disease is speedily developed.

*Occasional Causes.* Whether these be excitants or debilitants, whether the ordinary non-naturals in excess, or new agents, to which the body is not accustomed, whether, in producing irritation, they act by elevating too high, or depressing too low, the healthy vital actions; and whether they be naturally irritant or not, they are all capable of giving rise to the forms of disease in question. The longer and more violently they may have operated, the more readily and vigorously does the morbid form spring up, and still more so, if several causes of opposite natures have acted at the same time, or in rapid succession, as sudden alternations from heat to cold, from dampness to dryness, from motion to rest, &c. Upon any new and slight occasion, inflammation is liable to change into obstruction, congestion into inflammation, &c.

The proximate cause of these morbid forms, as long as they continue simple morbid *states,* consists in a mere change in the degree or impetus of the vital force: but, as soon as such morbid forms are established as *processes,* they consist in a change of mode of the vital action, which is invariably hyperstenic, whatever may have been the nature of the remote or exciting cause.

Brown considered morbid processes, as well as every thing else pertaining to life, as passive; and maintained that inflammation, obstruction, and congestion, were hyperstenic or hypostenic, according to the nature of their remote causes. Even the Brownonians, however, in what they term hypostenic inflammation, employ, under the name of excitants, the same resolvent remedies which the ancients, guided solely by experience, found advantageous. Our author's theory leads to the following practical maxim.

"All inflammations and obstructions are hyperstenic throughout the whole of their course; congestions, only when they exist as morbid states, may be hyperstenic or hypostenic, according as they arise from excitant or debilitant causes: but, even when they arise from debilitant causes, if they be established as morbid processes, or if they be changed into inflammations or obstructions, they immediately become hyperstenic."

Thus cold, grief, want of exercise may produce an afflux upon the heart, the liver or the brain; and, while the causes remain, and no more than a simple congestive state is manifested, it is admitted to be hypostenic, and to require motion, heat, joy and other excitants; but, should the congestive process remain, after the causes have been removed, or, should there be manifested an acute or chronic inflammation or obstruction of the heart, liver or brain, the disease must have become hyperstenic, and requires to be combatted by the antiphlogistic regimen, deobstruent and resolvent remedies, without any excitants.

Boerhaave regarded the proximate causes of these morbid forms to consist in an obstruction of the minute vessels of an organ; for which reason he made use of the word *obstruction*, as a general term to express the essence of these three distinct morbid forms, in the same sense as the modern Italian schools use the word *phlogosis*. Although Boerhaave's idea was derived from mechanical rather than vital laws, he succeeded better in practice than physicians of more modern schools, inasmuch as the living process in these obstructions of vessels, was considered as altered in mode as well as in degree. The ancients, while they employed resolvent remedies, were careful to reduce the strength as little as possible, whilst the moderns, in the treatment of *phlogosis*, from considering the vital process to be only elevated in degree, are led to observe no measure in reducing the strength of the patient. Our author's hypothesis, from admitting the invariable hyperstenia of these morbid forms, has an advantage over the Brownonian, and over the *contra-stimulant* doctrines, because it admits the moderation of the ancients in the use of reductive agents, since the living processes are allowed to be changed in mode, as well as in degree; whence it is evident that health cannot be restored by the mere force of reductive measures carried to extremes.

The strength or degree of hyperstenia in inflammation, obstruction, and congestion, is to be estimated by the obstinacy or pertinacity as well as by the violence of the affection. A disease does not change its essence from being acute or chronic, and the more chronic and obstinate a disease is, the more are powerfully resolvent remedies indicated and easily borne. Surgeons of the present day make a distinction in cases of wounds between those in which adhesion is prevented by excess, and those in which it is prevented by defect of inflammation; and if a part be hot, tense, painful and elastic, the inflammation is said to be excessive, but if the part be much swelled, soft, or dry and hard, but not very hot nor discoloured, the inflammation is said to be defective, because it is slow. Our author contends that, in the slow inflammation, obstinacy makes up for the want of violence; that an obstructed state or congestion of the surface of a wound always shews the disease to be serious, and that the importance of a disease ought to be estimated, according to the obstacle which it opposes to the restoration of health. The true adhesive inflammation is little entitled to be called inflammation, at least, its symptoms are always slight and transient, and every other inflammation whether acute or chronic, by which a wound may be attacked, must be hyperstenic.

The various species of inflammation differ from each other in mode, as well as in degree. Thus the true variolous pustule differs from the spurious, a phlegmon from erysipelas, erysiplas from carbuncle, carbuncle from a scirrhoid tumour, and this from an hæmorrhoidal congestion. In all of these diseases there exists hyperstenia, but the mode as well as degree of it differs in each.

The diagnosis of inflammations, obstructions, and congestions, are attended with difficulty when they are internally situated, and therefore not visible to the eye. It must rest upon the continued fixed pain, by which anatomy points out the part affected; upon its altered condition or consistency, if it be situated so as to admit of being felt with the hand through the external integuments; upon the state of its functions, which are liable to peculiar alterations in each disease of similar form; upon the fever; upon the alterations of colour and other sensible qualities; and, lastly, upon the absence of the signs of other diseases.

With respect to the prognosis, it is a general maxim in inflammation, obstruction, and congestion, as well as in other morbid forms, that when a due proportion is preserved between the constituent symptoms, redness, heat, pain and swelling, there is less danger than when any one of these symptoms greatly surpasses the rest in intensity. We have no room for extracting more of the author's observations on the diagnosis and prognosis of these affections. The following paragraph containing much of observation in few words seems, however, to deserve notice.

"If we carefully observe how much the vital functions are affected by the intensity, and by the disproportion between the symptoms of a disease; how much the functions of the affected part are influenced; and to what degree this influence acts upon the whole frame; and hence, according to the second general rule of the prognosis, if we regard the physiological state of the patient, the occasional causes, and the proximate cause of the disease, the importance of the organ affected, &c. it will not be difficult to ascertain the nature, whether evil or innocent, of any disease consisting in inflammation, obstruction, or congestion." 76.

In the second chapter of the second book, our author treats of fever, the theme of so many writers, and the stumbling-block of so many theorists; and of this affection, which, though so commonly the subject of daily observation, has divided the opinions of physicians in all ages, and in none, perhaps, more than the present, he gives a definition shorter, and, as it appears to us, more perfect, than any that has been attempted by preceding authors. It consists of two morbid characters only, namely, oppression in the exercise of the animal functions, and an augmented pulsation in the arteries, or an increased impetus in the exercise of the vital functions. If a man labours under both of these conditions, prostration of strength and augmented pulsation in the arteries, at one and the same time, he may be declared, without fear of mistake, to have fever. If, in some fevers, as the *algida* of Forti,

(which, we presume, to be the same as in this country is termed congestive fever,) the pulse appears less frequent than natural, yet it may be remarked, that its want of velocity is occasioned by the great oppression sustained, and also that it is still quicker than would appear proportionate to the extreme reduction of the powers of the body.

The particular symptoms of fever are arranged by our author into those of the *access*, those of the *height*, and those of the *decline*. In the access, the pulse is oppressed, and in proportion to the degree of oppression, it becomes quick, while, in the same proportion, are increased also the prostration of muscular strength, the thirst, the scarcity of visible excretions, the dryness and lividness of the skin, of the mucous membranes, of ulcers, and of the nails, the shrinking of the soft parts, the loss of heat, the sense of cold, the shiverings and rigors. When a fever is at the *height* the pulse dilates, and in proportion as it becomes full, its velocity is diminished: nevertheless the prostration of strength, the thirst, the defect of visible excretions, and the dryness of the skin and mucous membranes continue, but the parts previously livid, now become red, and we have an increase instead of a reduction of temperature, and a sense of heat, instead of a sense of cold. In the *decline* the diminution and disappearance of the above-mentioned symptoms take place, and, in proportion to their disappearance, is the humidity of diseased surfaces, and the abundance and thickness of excretions. Fever, besides the prostration of strength, which constitutes its chief feature, always produces a physiological debility, and an extraordinary waste of the substance of the solids and fluids. By the Brownonians, the prostration of strength, which is one of the elements of the physiological debility proceeding from fever, is confounded with it, under the term hypostenia. By this error, all fevers must be regarded as hypostenic, as soon as the patient appears to be much oppressed and enfeebled.

"An error more gross than this," observes our author, "certainly could not be made; because it is observed in every case, that no fever diminishes without the physiological debility being increased; and, in the convalescence, when the fever has disappeared, the weakness is greater than ever; whereas if Brown's view were correct, it ought to be quite the contrary." 79.

Fever may have a continued or an intermittent course. If continued, it presents the phenomena of the access at its commencement; those of the height more or less severe, but continued during all the days that the disease runs; and those of the decline at the termination of this. If the fever has an intermittent course, it presents the phenomena of the access, height and decline, returning from time to time, for many times in the course of the disease. The aggregate of the phenomena of all the three stages is a febrile paroxysm, and those fevers which have only one united paroxysm, from the beginning to the termination of the disease, are called continued fevers; while those which have several separate paroxysms are termed remittents, when, in the decline, the fever abates, but does not terminate, and intermittents, when, at the end of the decline, the fever so far terminates, that, between an antecedent and subsequent paroxysm, there is some interval of time, during which the disease appears to have ceased.

Intermittent and remittent fevers are called periodical, when the paroxysms return regularly at certain periods. Our author remarks that the being periodical is not peculiar to fevers, and that other diseases possess occasionally that form.

*Pathological Etiology.* The strong resemblance that exists between fever and inflammation is remarked upon by our author, observing that those who are most prone to inflammation are also most readily affected with fever. The united power of these two affections causes the death of the majority of the human race. Any cause capable of exciting inflammation of a part may, by acting upon the whole body, produce a fever: and there is no cause capable of producing a fever, which, if its action be concentrated in one part, produces any other effect, than inflammation. Any fever may be a predisposing cause, an occasional cause, a symptom, or an effect, of any inflammation, whether simple, congestive, or obstructive; and any inflammation may be a predisposing cause, an occasional cause, a symptom, or an effect, of any fever whatever.

Of the doctrine which assumes a diminution of the nervous energy to be the first element or proximate cause of fever, our author remarks, that this hypothesis has neither been logically maintained nor defended by its supporters, nor confuted by its opponents; because, supposing the first element of fever to be a diminution of the nervous energy, it is incumbent in the supporters of this opinion to shew, in what such a condition of the nervous system consists. He, moreover, denies that the remission of fever, proceeds from *atassia* (an injury) of the nervous fluid, or from *adynamia* (loss of power) of the nervous solids, or from hypostenia, and maintains that the affection peculiar to the nervous system, which is called a diminution of the nervous energy, consists in a phlogosis of the nerves: whence he infers, that even the slow nervous fevers are only slow neuritides.

Brown took the liberty of giving opposite significations to the two synonimous words pyrexia and fever, calling, by the former term, those fevers, in which the increased impetus of the arteries and of the physiological forces predominates, and designating as fevers, those pyrexiæ in which the diminution of nervous energy and physiological debility predominate. Here the loss of nervous energy is assumed without proof, as in the first mentioned hypothesis, and, this is added to the error peculiar to Brown, viz. the confounding the essence of the disease with that physiological debility, which is manifestly only an effect, and not a cause of the disease.

Boerhaave, as he considered inflammation to consist in obstruction of the capillaries of a part, so he regarded the essence of fever to consist in an obstruction of the capillaries generally throughout the body. Thus he regarded fever and inflammation as morbid forms of the same nature. This theory, although it is founded upon mechanical laws, has the same advantage over the Brownonian in the treatment of fever, as it has in that of inflammation.

Our author regards fever and inflammation as of the same nature, and says that while fever may be said to consist in a phlogosis generally diffused throughout the whole body, an inflammation may be said to be a fever concentrated in one part. He makes a distinction between a febrile state and a febrile process, saying,

"1. That the debilitating remote causes do not immediately produce more than a congestion generally diffused, which, from causes internal or external, being changed into fever, the result is always hypersthenic: 2. That the exciting causes, such as heat, running, spirituous liquors, produce a violently heated state; which, although it be not commonly called fever, yet is in fact an hypersthenic febrile state. 3. That the irritative causes, in producing a fever, do nothing else than produce a phlogosis throughout the body, of an hypersthenic nature: and, 4. That, whatever may have been the remote causes, and, however they may have operated, if the universal phlogosis be already established as a process, the fever that results is hypersthenic, as every inflammatory process is." 84.

He draws arguments in proof of the constantly hypersthenic nature of fever, from the identity of its causes with those of inflammation. Maintaining that diminution of the nervous energy is a symptom of phlogosis of the nerves, he reserves the demonstration of this fact till he comes to treat of nervous affections. The loss of flesh sustained by patients in fever, he attributes to the phlogosis of the excretory vessels, and the proof of this he reserves for his discussion on the profluvia or fluxes. He conceives that an irrefragable proof of the hypersthenic nature of fever is afforded by considering what use a person in fever instinctively makes of the non-naturals or external things. Thus, he leaves off all the ordinary stimulants which he can dispense with, and helps himself sparingly to such as are either absolutely or from long custom necessary. He asks for aqueous drinks; refuses spirituous liquors; food, unless it be thin or fluid, is always heavy and difficult to be borne, and hurtful in its effects; he is averse to exercise; derives relief from lying in bed, and encouraging perspiration; he desires sleep, and often dislikes the stimulus of light, withdrawing himself from it. A regimen the reverse of this is hurtful, in all cases without exception.

Even the Brownonians regulated the treatment of their supposed hyposthenic fevers in this manner, but then they regarded all medicines as stimulants, and absurdly combined remedies of the most opposite natures in their prescriptions. In reply to the question, if fevers be always hypersthenic, how does it happen that bark, a tonic remedy, cures an intermittent, our author says that it affords him one of the strongest proofs of the hypersthenia, because the bark invariably proves hurtful, when given during the paroxysm, which can be successfully treated only by the reductive or resolvent method. In the cure of intermittents, bark operates as a prophylactic fortifying the system to resist the attack of the paroxysms. On the other hand, he asks those who contend for the hyposthenic nature of fever to explain how it happens that bark is hurtful during the paroxysms, and how the reductive regimen proves beneficial.

Our author remarks that the result of the treatment affords a proof of the hypersthenic nature of fever, since, although, by some anti-Hippocratists, the use of stimulants in fevers has been often introduced, and they may have boasted of cures obtained by that method, yet the Hippocratic or antiphlogistic method has always been triumphantly resorted to, and followed by the most accurate observers, that is, by those who guided their practice by observations of facts, rather than by theoretical speculations. The object of this method is not to torture the sick by force of privations and evacuations, but to use reductive means prudently, so as to diminish the fever, as much as possible, and to reduce the physiological strength as little as possible.

That the phenomena of fever are invariable in all cases although different patients, or the same patient in the various stages of the disease, may be in different states, as to vigour or debility, our author considers as a further proof of the hyperstenic nature of the disease. Moreover, even in the supposed hypostenic fevers, when the fever abates, the strength does not cease to diminish; and during convalescence, when the fever is at an end, the debility is not unfrequently greater than ever, which shews that the nature of the disease was hyperstenic, notwithstanding the physiological debility of the patient.

The danger or severity of a fever is in proportion to the degree of prostration of strength, and to the physiological debility or disturbance of the animal functions. Alienation of the senses is a worse symptom than simple delirium; excessive physiological debility, is worse than excess of vigour; symptomatic fluxes than retention of excretions; lividity than bright redness; lowness of temperature, than great heat; and smallness, straitness and irregularity of the pulse, than great fulness, tension, hardness and quickness.

In the third chapter of the second book the resolution and the changes of nature as well as of place, to which fever, inflammation, obstruction, and congestion are liable, are treated of, under the heads *phenomenology*, *pathological etiology*, *diagnosis* and *prognosis*. As the author's observations on this part of the subject seem to be the result of accurate observation and reflection we shall attempt to give a condensed view of them.

Every disease may terminate by resolution, when health is the result; by conversion, when it changes into another disease; or by death. By conversion, a disease may change its seat alone, or its form alone, or both at the same time. When a disease changes its form, it may take another analogous form, as when inflammation changes into obstruction, congestion into inflammation, &c. or it may degenerate into a form altogether different, as when inflammation terminates in the destruction of a part, or in morbid growths, &c.

The resolution of any disease is manifested by the patient feeling better, and being relieved from oppression, but it is the duty of a physician not to mistake a fallacious sense of relief which sometimes is only the harbinger of death, for that real relief which indicates resolution. When resolution actually comes on, all the phenomena of the disease gradually and progressively subside, as soon as the disease has completed its proper course. In proportion as the pathological vital actions decline in vigour, the physiological debility becomes more apparent; and, when the physiological strength and the flesh begin to recruit, the resolution may be considered as certainly established. If the disease has been accompanied with a symptomatic flux, it is observed gradually to abate as the resolution is established; and, if any unusual dryness or stiffness has existed, the resolution is accompanied with a reappearance of the suppressed secretions. The secretions or excretions which denote a true resolution, ought to occur in the situation, at the time, in the quantity, and of the kind required by the nature of the disease.

The conversion of a disease is recognized by its not ceasing when the time for resolution is past; by the disease appearing to decline, but not so regularly or effectually as in resolution; or by its becoming unseasonably much aggravated; and by the signs of the new form or new seat which the disease threatens to take, already beginning to shew themselves. With respect to changes of form, fever, inflammation, obstruction and congestion are each convertible into the others. With respect to changes of seat, it is distinguished into two kinds, diffusion and translocation. The former consists in a morbid form not quitting its original seat, but propagating itself, and seizing upon organs akin or near to it, or proceeding to affect the whole body: thus, a fever may diffuse, in some organs, inflammation; in others, congestion; and in others, obstruction: an inflammation may diffuse a fever throughout the system, or it may give rise to inflammations, obstructions or congestions in the neighbouring organs. The same effects may proceed from obstruction or congestion. Translocation, or metastasis consists in a morbid form quitting entirely the organ or part where it was originally seated, and being translated under the same or a different aspect to another seat; as when fever terminates in a local inflammation, or when inflammation disappears, gives rise to a fever, &c. A disease sometimes passes from an external seat into an internal organ: this change is called *retro-pulsion*: if, on the contrary, a disease quits an internal organ and seats itself externally, the change is termed *expulsion*.

With respect to the causes which predispose to the resolution or conversion of diseases, they must reside in the constitution of the patient, for, in proportion to the strength and healthiness of the constitution is a disease prone to resolution in preference to conversion either of form or seat, whether the disease be in a morbid state or a morbid process. A morbid process, however, has a necessary course to run, and whether its termination shall be in resolution or conversion must very much depend on the nature of the disease, because, although a morbid process occurring in a strong constitution, and properly treated, more frequently terminates in resolution than in conversion, yet it may be converted, while the same disease occurring in a bad constitution, may end in resolution, notwithstanding improper treatment. This piece of pathology, which is as old as it is true, was recognized by the ancients in their distinction of diseases into critical and non-critical. Morbid states are non-critical because their events depend on the means employed to remove their causes, and on the goodness of the patient's constitution. Morbid processes are critical, that is, subject to a decisive change at a given time, which depends chiefly on the intrinsic nature or quality of the disease.

For this reason, when endeavouring to ascertain whether the means employed in fever, inflammation, obstruction, or congestion, when established as processes, have been useful or hurtful, not one case in particular must be relied upon, but we must either take the average result of many similar cases, or calculate whether, in the course of the cure, the particular effect of individual remedies has been an alleviation or an aggravation of the symptoms.

As to the occasional causes of resolution, it can only be produced by excitants in hypostenic affections, and by reductive agents in all other cases. To ensure success the proper remedies must be given in due time and suitable doses. A slow and pusillanimous practice, by omitting the necessary means at the seasonable time, may permit the conversion of a disease, which, with proper management, might have terminated in resolution. On the contrary, by employing relaxant remedies harshly, they may act as irritants. We ought to adhere to the maxim of the ancients, that a remedy ought at once to be efficacious and bland in its operation; or, in the words of our author's rule, relaxant or reductive means ought never to be different in kind nor less in degree than is required by the violence of the disease, while they ought to be such as can be borne with ease by the patient.

Irritative causes are, under all circumstances, hostile to the resolution of diseases, and frequently efficient causes of conversions as well as of degenerations.

As to the proximate cause or essence of resolution, it consists manifestly in a regular and determinate declension, in the degree or impetus alone of the vital force, or both in the degree and mode in which that force operated during the disease. The proximate cause of conversion is a change of the vital action from one degree of disease to another degree, also morbid, or from one degree and kind of disease, into other degrees and kinds, also morbid.

With respect to the prognosis in the various terminations here discussed, resolution is always the most desirable, as it is the most fortunate; but, though conversion, as compared to resolution, be a less fortunate termination, yet, as compared to death, to conversion into a worse form, or to translocation to a more important organ, it may be desirable. Every *expulsion* is advantageous, and every retropulsion hurtful. The conversion of a morbid form into another more inclined to resolution is advantageous, and the reverse hurtful. Moreover, in some diseases which are not susceptible of a radical cure, the best resource of art is to procure a favourable transmutation, either with regard to their seats or forms.

The general cure of fever, inflammation, congestion and obstruction, forms the subject of the fourth chapter of the second book; and, in the first place, the *specific cure* is considered. In scabies, syphilis, herpes, scrofula and rachitis, sulphur, mercury, antimony and iron, do not operate as excitants, but as resolvents, and, accordingly in fevers, inflammations, congestions, and obstructions of a scabby, herpetic, scrofulous and rachitic origin, they act, according to our author, at once as specifics, cancelling the peculiar qualities of such diseases, and generally as resolvents by diminishing the degree of such morbid forms. These medicines may, therefore, be employed with safety, even in affections unconnected with the diseases in question, since, although there may be no opportunity for them to act as specifics, they may still be useful as general relaxants or resolvents. Even when they may be employed as specifics, it is necessary to use them with such moderation as to prevent their proving irritants.

The *minorative cure* is had recourse to, when there are no specific means, and its object is to abate the impetus of inflammation, obstruction, or congestion. With respect to a fever we have to determine when the means ought to be excitant, and when debilitant, and in the latter, what debilitant remedies are to be employed and in what manner. Congestions while they continue mere morbid states dependent upon debilitating causes, are to be treated by an excitant method, but in all other cases either of hypersthenic congestions, or of congestions which having originally been hyposthenic have already become morbid processes, or of inflammations, obstructions, or fevers, in whatever manner originally established, the method of cure ought to be relaxant.

In discussing the propriety of employing the strong resolvents or relaxants in the cure of fever, inflammation, obstruction, or congestion, our author says, that a metallic solution or the distilled water of lauro-cerasus, applied to a phlegmon, frequently act as irritants; whereas, if on the first attack of phlegmon, it has been treated by means of bleeding and emollients, the former remedies then act beneficially; or, if applied to inflammation of a slow, obstructive, or congestive character, they are borne without inconvenience, and exert their regular relaxant or solvent power. We believe that the lauro-cerasus is not used in this manner, nor for this purpose in these kingdoms. The author goes on to remark that those actions and changes which take place visibly before our eyes in external diseases, must be admitted to take place also under the use of medicines in internal diseases, and that from the former we may learn how to direct our practice in the latter.

Another observation, so far as we know, seems peculiar to the author. If there has been a debilitating agent among the occasional causes of a fever, inflammation, obstruction or congestion, he maintains that we can never employ the same agent, to bring about resolution of the disease. For example, if emetic tartar has had a share in the production of gastritis, we may avail ourselves of every other reductive or relaxant remedy in the treatment of the case, but never of emetic tartar. Or, if cold has had a share in producing pneumonia, we should never resort to the use of cold for its cure. He says he is aware that apparent exceptions might be adduced, but that such cases admit of explanations for which he refers to a subsequent part of his work.

With respect to the extent to which relaxant or reductive means ought to be carried, our author remarks that, when we have no specific remedy, we can do good only by subtracting from the physiological strength so as to diminish the impetus of the pathological actions; and, although a prudent physician considers it his duty to husband the physiological strength of his patient, yet he will not fear to repeat both bleeding and relaxant or resolvent remedies, as long as they are required by the violence of the disease, being well persuaded, that *one ounce of blood too much may kill a man, but that no man will die for a pound too little.* We have placed these important words in italics, as they seem to us to shew, in an incontestible manner, the soundness of the doctrines peculiar to M. Lanza, since they lead to the same practical result in the most important class of diseases, that has been arrived at, under the guidance of experience rather than of theory, by the best physicians in this country. Should a disease, perchance, be unsusceptible of resolution, or if, from being slight, no fear of death need be entertained, or if the physiological strength may have been sufficiently reduced already, it is proper, our author remarks, even while reductive means are employed, to allow food, although sparingly.

Great attention ought to be paid, in the cure of diseases to the particular idiosyncrasies of patients, because it is not uncommon to meet with individuals who will bear conium, squill, or mercury, yet not digitalis or antimony: hence, the ordinary medical attendant of a patient is not preferred without reason, since he must be better able than any one else to pursue a method of cure that shall prove efficacious, while, at the same time, he avoids causing irritation.

Thus, the new Italian medical doctrine, by admitting the invariable hyperstenia of morbid processes, leads, like the modern and most ancient schools, to the relaxant or reductive method: while, by the theory of changes of mode, which belong to morbid living actions, the abuse of reductive agents is restrained, and the practice deduced from these principles is the same that Hippocrates, from long experience, deemed the most advantageous.

In treating of the *prophylactic cure*, our author enquires whether bark ought to be esteemed a febrifuge, or an anti-periodical. He decides that it is the latter for the following reasons:—

"The ascertained facts relative to this question are, 1. That the bark only prevents the return of periodical diseases, so that, if employed in diseases, which, though intermittent, are not of the true nature of periodicals, as in the spurious intermittents so called, it does no good, and often harm. 2. That the bark interrupts the course of periodical diseases, whatever be their form, whether from inflammations, nervous affections, or fluxes, &c. 3. That the bark, administered in the paroxysms of periodical diseases, not only does no good, but harm, and, therefore, must do harm in continued diseases. 4. That, during the paroxysms of periodical diseases; the relaxant method is borne without inconvenience, and indeed is useful: in fact, even the Brownonians, in the paroxysm of a periodical disease, do not hesitate to employ debilitating means, and at times even blood-letting." 104.

Bark is beneficial, not only in fevers, but also in inflammations, nervous diseases, and fluxes, when they are periodical. Hence arises the question, how does bark subdue periodical diseases? Two opinions may be formed on this question; that fevers and periodical diseases are hypostenic, and that bark acts as a stimulant. In this way it is impossible to explain why it is hurtful during the paroxysm; and, again, why the reductive method of treatment, consisting of cool drinks, the acids, fasting, keeping in bed, and the loss of blood are beneficial. The other opinion is, that morbid processes, though periodical, do not cease to be as much hyperstenic as if they were continued, and that the bark obscures the periods of such diseases, as opium lulls their pain, although they may be hyperstenic. This opinion, not general in the present day, and almost obsolete in the schools, is adopted and maintained by our author.

With respect to the *palliative cure* of irritative fevers, inflammations, obstructions and congestions, our author remarks that the physician can do no more than attempt to palliate symptoms, until Nature, the art of surgery, or time, shall have applied the specific cure, by expelling or extracting the cause of irritation, or rendering it habitual and no longer troublesome. This palliative cure consists in moderately subtracting the ordinary stimulants, observing an antiphlogistic regimen, using sparingly some remedy of a relaxant or reductive kind. This reductive method is to be increased in energy as the increasing violence of the disease may require. In all cases palliative means ought to be as efficacious as the nature of the disease requires, not more violent than the physiological strength can bear, nor employed in such a manner as to prove irritant. Our author further lays down strict rules for regulating the use of palliatives when they consist of remedies of a nature contrary to those required for the radical cure of the disease, as when opium is employed to ease pain, or tonic astringents to restrain a flux, a method of cure which, as he justly observes, requires the utmost caution and circumspection, as such remedies are extremely apt to prove irritant, unless they have been duly preceded by evacuants and emollients. In proportion to the acuteness or violence of the disease is the danger to be apprehended from such remedies. It is likewise particularly necessary to beware of them when a morbid process seems to be threatening to arise. In proportion as the symptoms requiring the use of excitants are essential to the nature of the disease, it is hurtful or dangerous to employ them. They can only be used safely against pains, which are adventitious or dependent on a cause totally different from the principal disease.—For example, laudanum may be given to soothe a colicky pain in a patient labouring under a fever, provided the pain be not the effect of the fever, but of some adventitious cause, and provided the fever be of such a nature as not to suffer from the laudanum; but for a cough, the necessary effect of a pleurisy, it is never allowable to use opium.

With respect to revulsive or revellent agents, as blisters, &c. our author maintains, that, although they excite inflammation of the skin or cellular membrane, they really act as solvents or reductives with regard to the internal organs affected with inflammation, obstruction or congestion, and that the hyperstenic nature of such affections, or of fever, is no valid objection against their use, when care is taken not to apply them in the first impetus of an acute disease.

The fifth chapter of the 2d book is devoted to the consideration of nervous complaints. Our author reduces their phenomena to five heads, viz. *pain*, *stupor*, *convulsion*, *torpor*, and *error*. We have not space to admit of our going at length into his interesting observations upon this subject. To show how far the phenomena of nervous diseases depend upon their proximate causes, he adduces the following practical facts.

"1. Under the immediate action of any agent whatever, whether stimulant, debilitant, or irritant, there may arise pain, convulsion, stupor, torpor, and error: the poisons afford the most frequent examples of this. 2. During the same disease, and at the same time, in some organs, may be manifested pain and convulsion, and in others, stupor or torpor: this is seen in an epileptic paroxysm. 3. It is ordinarily the case that pain is conjoined with convulsion, and torpor with stupor; nevertheless, worms often occasion violent convulsions without pain; and it is not rare to observe, on some occasions, torpor without stupor, or even with increased sensibility. 4. In error, some sensations become preternaturally acute, and others are stupefied at the same time, and like discordance appears in the judgment. 5. Finally, it is observed, that there may be manifested in the same part, alternating, from hour to hour, pain and stupor, or convulsion and torpor: the *coma vigilans* is a manifest example of this. To express these facts in general terms, we say—*That a nervous disease, from the variation of some circumstance, of which, in practice, we cannot calculate either the form or the cause, may manifest either of the five morbid phenomena peculiar to it, and display them in any combination or alternation.*" 117.

We have not room to examine at any length the arguments which the author advances in support of his opinion that nervous diseases are essentially phlogoses of the nerves. The principal arguments are the convertibility of nervous diseases into inflammations, fevers, or other morbid forms proved or admitted to be of a hyperstenic nature, and the fact that persons affected with nervous diseases are obliged to observe the most abstemious regimen, and to submit to the most rigid privations with regard to the most common stimulants, which not only cause inconvenience, but are intolerable to them. Morbid anatomy shews in the bodies of those who die of nervous diseases, either the brain, the medullary nervous pulp, the ganglia, the nerves or the muscles, tumid and full of blood or sanguinolent lymph, which are marks of congestion; sometimes red and hard, that is inflamed; sometimes cineritious, dry and hard, that is, obstructed.

In the 6th chapter, the subject of a bad habit of body is discussed at considerable length, and in a very interesting manner. The following passage shewing the resemblance between the doctrines of Professor Lanza and the humoralist pathology seems curious,

"The resemblance between our modern and the ancient humoralist pathology is to be seen, if we keep in mind the value of the obsolete term *morbific humor;* since this, by serving as a general formula to indicate the proximate cause of all morbid forms, febrile, inflammatory, obstructive, congestive, nervous, &c. signifies what, in the modern schools, the word *phlogosis* imports, employed in like manner as a general formula to indicate the essence of the morbid form itself. And therefore we shall see, in due time, how the dyscrasia lymphatica, biliosa, sanguinea, and atrabiliosa of the ancients correspond to phlogoses diffused in the arteries or the veins, or in the lymphatics or in the nerves; and the terms *hot humor, slow humor, fixed humor,* &c. we shall find evidently to correspond to these of the moderns, phlogosis *calida, lenta, fixa, vagans,* &c. From this it seems, that if, in reading the classical works of the humoralists, we change the word *humor* for that of phlogosis, it will almost appear that the ancient pathology is the modern, and the modern will seem as old as medicine itself; for example; in the gout, the ancients admitted a *gouty humor,* latent and spreading in the body, for the most part residing in the lymph, which, by manifesting itself at given times, produced the gout regular or irregular: we say, that in the gout there is a change of quality in the vital power, which produces a continual process of inflammation *(phlogosis),* which, for the most part, is diffused in the lymphatics, and here it is hidden, until, kindled at certain times, it is manifested by that inflammation, regular or irregular, acute or chronic, in which consists the gout." 146.

Passing over the chapter on bad habit of body, which will be found to afford much interesting matter for reflection, as well as that on the morbid alterations of the temperature, excretions and colour of the body, either of which our limited space forbids our attempting to analyse, we extract from the chapter on wounds and the healing process, the following passage, because the peculiar opinion which the author advances respecting what is termed the adhesive inflammation seems to be well founded, though certainly at variance with that which has been generally received by pathologists, since the time of Mr. Hunter.

"Is it true, that, for the healing of wounds, a certain inflammation, called adhesive, is necessary? From observing that cold-blooded animals are less liable to inflammation than those of hot blood, and that they have the faculty of forming new parts in greater perfection; that in our bodies, the parts least liable to inflammation, as the bones, have a stronger power of regeneration than others; that the wounded, who are left ensanguined on the field of battle, recover better than those assisted by surgeons, who are often more officious than informed; that it is never the greatness, but rather the smallness, of the quantity of blood lost that prevents adhesion; and that what is termed the adhesive inflammation, is the more efficacious in proportion as it is less considerable, and of short duration; we are persuaded that the healing is entirely the work of the natural vital force, not irritated nor inflamed, but healthy. But, from our having distinguished inflammation into a morbid process, and a simple morbid state, the present question may be settled with the greatest facility and clearness: because, it not being possible that a true morbid inflammatory process can supervene upon parts, when wounded and separated, without suppuration taking place, it follows, that the adhesive inflammation cannot be believed to be any thing but a simple morbid hyperstenic *state.* Now, it being inevitable that the wounded substance must suffer irritation and hyperstenia, from the new agents with which it comes into contact, it would be a needless dispute to maintain, either, according to our view, that the irritation and state of phlogosis, although always hurtful, yet admit, when they are slight, of the natural power of uniting the sides of a wound, or, according to the contrary opinions, that such morbid state of phlogosis and irritation are also those salutary means, by which the vital force performs the sublime office of re-uniting wounds, if it were not that two ideas, pernicious in practice, have arisen from the hypothetical idea of adhesive inflammation, the instant correction of which we cannot help demanding of surgeons. 1. That the increased vigor of the machine, and the hyperstenia of the part, is believed to be very useful to the re-union of wounds. We, on the contrary, believe that, for the re-union of wounds, it is always required that the irritation and phlogosis of the part, if it were possible, should be reduced to nothing, and that in all cases there ought to be induced so much physiological debility as to remove every disposition or cause of any threatened irritation or inflammatory process. 2. That the healing may fail, either from excess or from defect of inflammation. On the contrary, we have demonstrated, that in cases of violent irritation, with stupor and torpor, from contusions and other accidents, and from obstructions, congestions, callosity, &c. of the wounded part, there does not exist debility, but the irritative or inflammatory state, more severe than ever, whence the relaxant method becomes the more useful." 184.

Our author's views respecting suppuration, and especially the distinction he makes between that inflammation which precedes, and that which accompanies this process, as well as his observations on the resolution and degeneration of suppurations, when once established, are also peculiar, and are manifestly the result of much careful observation and profound thinking in an original manner on the phenomena in question.

We are here compelled, however, to close our remarks on this volume, which, we hesitate not to declare, will be found replete with novel ideas and no small portion of valuable information. It would be a good exercise for a student entering upon the career of his professional duties to train his mind in the methods here pointed out for investigating diseases, and thus to form early habits of not yielding his assent to the dicta of any authority without being sensible of the deliberate conviction produced by sound reason upon his own mind.

A new general system of medicine, which, notwithstanding the author's modesty, is unquestionably developed in the pages of this volume, is a curiosity, as well as a novelty in the present state of medical literature; but we apprehend, that the work will be admitted to possess the more rare merit of simplicity, and of gaining the general assent to the doctrines unfolded in it. From this specimen of the author's general pathological principles, we should rejoice in an opportunity of seeing his method of applying them to individual or particular diseases. The translator, however, has not furnished us with that part of the work on the present occasion.

There are several mis-translations, which we deem it our duty to notice, and which would almost lead us to suspect that the translator must either be a foreigner, or must have resided so long abroad as to have acquired the habit of thinking in a foreign language. For example, we find the word "*burning*" employed where it is evident from the context, that the "*being over-heated*" was meant. In like manner we have "*molestation from cold,*" to express the state of a person suffering under the effects of extreme cold; "*exhaustion from tiresomeness,*" instead of *exhaustion from fatigue*: "*cold to the burned*" to signify the reduction of the temperature of a person who is overheated; "*the which*" instead of *which*. Such errors which greatly disfigure the translation, and sometimes obscure the sense, are much to be regretted, but those who take that interest in the subject matter which it really deserves, will not be deterred by them from carefully perusing the volume.

